# Redox control in the pathophysiology of influenza virus infection

**DOI:** 10.1186/s12866-020-01890-9

**Published:** 2020-07-20

**Authors:** Ker-Kong Chen, Moeko Minakuchi, Kenly Wuputra, Chia-Chen Ku, Jia-Bin Pan, Kung-Kai Kuo, Ying-Chu Lin, Shigeo Saito, Chang-Shen Lin, Kazunari K. Yokoyama

**Affiliations:** 1 School of Dentistry, Kaohsiung Medical University, Kaohsiung, 807 Taiwan; 2 Department of Densitory, Kaohisung University Hospital, Kaohisung, 807 Taiwan; 3 Waseda Research Institute for Science and Engineering, Waseca University, Shinjuku, Tokyo, 162-8480 Japan; 4 Graduate Institute of Medicine, Kaohsiung Medical University, 100 Shih-Chuan 1st Rd., San-Ming District, Kaohsiung, 80807 Taiwan; 5 Regenerative Medicine and Cell Therapy Research Center, Kaohsiung Medical University, Kaohsiung, 807 Taiwan; 6 Department Surgery, Kaohsiung Medical University Hospital, Kaohsiung, 807 Taiwan; 7 Saito Laboratory of Cell Technology Institute, Yalta, Tochigi, 329-1471 Japan; 8 Department of Biological Sciences, National Sun Yat-sen University, Kaohsiung, 80424 Taiwan; 9 Cell Therapy and Research Center, Kaohsiung Medical University Hospital, Kaohsiung, 807 Taiwan

**Keywords:** Antioxidation, Aryl hydrocarbon receptor, Cellular oxidation, Nuclear factor E2-related factor 2, Reactive oxygen species

## Abstract

Triggered in response to external and internal ligands in cells and animals, redox homeostasis is transmitted via signal molecules involved in defense redox mechanisms through networks of cell proliferation, differentiation, intracellular detoxification, bacterial infection, and immune reactions. Cellular oxidation is not necessarily harmful per se, but its effects depend on the balance between the peroxidation and antioxidation cascades, which can vary according to the stimulus and serve to maintain oxygen homeostasis. The reactive oxygen species (ROS) that are generated during influenza virus (IV) infection have critical effects on both the virus and host cells. In this review, we outline the link between viral infection and redox control using IV infection as an example. We discuss the current state of knowledge on the molecular relationship between cellular oxidation mediated by ROS accumulation and the diversity of IV infection. We also summarize the potential anti-IV agents available currently that act by targeting redox biology/pathophysiology.

## Background

Influenza viruses (IVs) have been involved in pandemics and seasonal epidemics and are serious threats to humans [1–4]. IV pandemics have been noted in the past; for example, the Spanish flu caused by the H1N1 IV in 1918, the Asian flu caused by the H2N2 IV in 1957, the Hong Kong flu caused by the H3N2 IV in 1968, the bird flu caused by the H5N1 and H7N9 IVs in 2003 and 2013, respectively, and the swine flu caused by the H1N1 IV in 2009. Vaccination is currently the key strategy against IV infection, although this strategy is limited by the ability to produce effective vaccines that precisely target new and emerging IV strains.

Three drugs (zanamivir, oseltamivir, and peramivir) are structurally related and inhibit neuraminidases on the cell surface membranes of both influenza A and B viruses and are approved by the US Food and Drug Administration [5]. Both amantadine and rimantadine can target the IV M2 protein, to inhibit the proton channel [6, 7]. Baloxavir marboxil is a selective drug that inhibits the IV cap-dependent endonuclease, thus preventing IV proliferation by inhibiting the mRNA initiation reaction [8]. However, resistance to anti-IV drugs is a major problem associated with these well-known therapeutics' agents; moreover, the safety of some of these agents in pregnant women and fetuses is not well defined [9].

Reactive oxygen species (ROS) are often produced during IV infection [10], thus promoting apoptosis, lung injury (LI), and inflammation/allergy [10–13]. These studies indicate the crucial roles of ROS in IV infection, which may have implications for therapy. In this review, we summarize ROS generation and redox control of the host cells upon IV infection and discuss how ROS can affect IV replication. We also describe the potential therapeutics against IV infection that targets ROS prevention and antioxidation in host cells. In addition, therapies aimed at inhibiting ROS-mediated cell damage are critical for future use. Thus, here we show the advantages and disadvantages of these anti-influenza drugs from new perspectives.

## Main text

### ROS generation and antioxidation system in cells

Mitochondria are the target organelle of oxidation–reduction reactions [14–16]. Mitochondria play major roles in the production of both adenosine triphosphate and ROS during oxidative phosphorylation [15]. The hydrogen peroxide (H_2_O_2_), hydroxyl radicals (^●^OH), and superoxide anions (O_2_^–^) that are generated mainly during oxidation are classified as ROS [14, 16]. Reactive nitrogen species (RNS) are ROS that contain nitrogen. Based on their chemical properties, ROS/RNS have been divided into two groups: free radicals and non-free radicals. Free radicals are ROS that contain one unpaired reactive electron in the outer orbit and include one-electron oxidants, such as nitric oxide (NO), carbonate radical anion, nitrogen dioxide, alkoxyl/alkyl peroxyl, ^●^OH, and O_2_^–^. The non-free radicals do not contain unpaired electrons and include two-electron oxidants, such as peroxynitrite, peroxynitrous acid, hypochlorous acid, singlet oxygen, and H_2_O_2_. Free radicals are unstable and more reactive than non-free radicals. ROS/NRS target molecules that contain protein residues such as Cys, Met, Tyr, and Trp, and iron-sulfur (Fe-S) bonds. The targeting of ROS to macromolecules such as DNA, RNA, proteins, and lipids leads to various biochemical and pathological alterations [16].

Intracellular ROS are produced mainly by enzymes, such as those in the mitochondrial nicotine adenine dinucleotide phosphate (NADPH) oxidase (NOX)/dual oxidase (DUOX) family [17], xanthine oxidase (XO) [18], cytochrome p450 (CYP) [19], polyamine and amine oxidases (or polyamine-catabolizing enzymes) [20], and lipid-catabolizing enzymes [21]. The mechanisms underlying the production of O_2_^–^ and the single-electron reduction of 8-nitroguanosine to the respective anion radical via NADPH–cytochrome P450 reductase (POR) have been reported [12]. 8-Nitroguanosine is often produced in inflamed or infected tissues [12], and its derivative, 8-nitroguanosine 3′,5′-cyclic monophosphate (8-nitro-cGMP), is also a functional molecule. The metabolic pathways of 8-nitro-cGMP are produced by the inducible nitric oxide synthase (iNOS) enzymes, which react with SH-proteins via S-guanylation, to form 8-SH-cGMP by reacting with endogenous hydrogen sulfide/sulfanyl radical H_2_S/HS^●^ [22]. Any of the iNOS enzymes, or even XO, can subsequently transfer electrons to molecular oxygen [23].

The redox homeostasis is maintained in normal cells by the antioxidation reactions, which include the antioxidant systems of several enzymes, such as superoxide dismutases (SODs), catalases (CATs), peroxiredoxins (Prdxs), glutathione peroxidase (GPx), and glutaredoxins (GRs), and a nonenzymatic system, which consists of anserine, carnosine, carotenoids, flavonoids, glutathione (GSH), homocarnosine, melatonin, and vitamins C, and E [24, 25]. The decrease in GSH/glutathione disulfide (GSSG), which is a cellular antioxidant index, is caused by the reduction in the level of GSH, which acts as a redox buffer within cells [25]. GSH is capable to protect the damages to intracellular components caused by ROS such as free radicals, peroxides, lipid peroxides, and heavy metals. It also plays a critical role in suppressing oxidative stress in several RNA viruses, including IVs [26].

A comparison of the kinetic data of H_2_O_2_ metabolism between thiol targets and GSH has been performed in mammalian cells. The decomposition of H_2_O_2_ by GSH is dependent on the concentration of H_2_O_2_, and GSH has a lower affinity for H_2_O_2_ than do GPx and Prdx. Therefore, both GPx and Prdx are the main metabolic pathways for H_2_O_2_ at low concentration, whereas GSH would have a prominent function at higher concentrations of H_2_O_2_ [27]. A kinetical analysis has shown that both Prdx2 and GPx1 are strong sensors of H_2_O_2_ [27].

The nuclear factor E2-related factor 2 (Nrf2) controls the expression of enzymes that participate in the defense against oxidation, although many Nrf2-independent ROS clearance enzymes and nonenzymatic antioxidants also exist [28]. In the presence of normal levels of ROS, the expression of Nrf2 is controlled by the Kelch-like ECH-associated protein 1 (Keap 1), which targets Nrf2 to ubiquitin-mediated degradation in the cytoplasm. In the presence of increased ROS production, the Nrf2 molecule dissociates from Keap 1 and moves into the nucleus, where it binds to the antioxidant-response element (ARE), together with the small MAF transcription factors, in the promoter regions of target genes that encode antioxidant enzymes. Notably, the promoter of the *Nrf2* gene itself contains AREs [29] and amplifies the redox cascades via positive-feedback regulation in cancer cells or phase I ligand-responsive cells.

The cytoplasmic aryl hydrocarbon receptor (AhR) protein binds to the ligands that have translocated into the nucleus and activates the expression of a large family of antioxidant molecules, i.e., the cytochrome p450 proteins (CYP1A1, CYP1A2, and CYP1B), in cancer [30], as well as several other antioxidation molecules, such as NAD(P) H quinone oxidoreductase 1 (NQO1), after the formation of heterodimers with Arnt. The AhR-dependent increases in neutrophilia and iNOS levels in the infected lung were reported to be mediated by AhR-regulated events extrinsic to bone-marrow-derived cells [31, 32]. An experiment using Cre/*loxP* technology confirmed that AhR-mediated iNOS increases and neutrophil migration to the lung during IV infection [33].

### Influenza virus (IV)

The genomes of IVs consist of negative single-stranded RNAs that are associated with the viral nucleoprotein (NP). They interact with viral RNA-dependent RNA polymerases heterotrimer, i.e., the polymerase basic protein 1 (PB1) and 2 (PB2) and polymerase acidic protein, to build the viral ribonucleoprotein (vRNP) complexes.

Human influenza A virus (IAV) infections generated pandemics in 1918 caused by H1N1, in 1957 by H2N2 and in 1968 by H3N2 [34]. Pandemic IVs cause much higher morbidity and mortality than outbreaks of annual and epidemic IVs. IV infection includes both upper and lower respiratory tract involvement. IV pneumonia resulted in either alone or with secondary bacterial pneumonias. The IV pandemic in 1918 known as worst pandemic on record indicated the death of up to 50 million people globally. However, it was also reported that the spectrum of pathologic alterations shown in the 1918 IVs pandemic does not differ from those of pathological abnormalities on other less-pandemic patients or even in dead patients during seasonal IV outbreaks [35]. One exception is the hypercytokinemia or cytokine storm; one of the possible features proposed to explain the pathogenesis of H5N1 pandemic infection [36].

The PB1-F2 protein is expressed from the PB1 gene of most IAVs, which is localized in mitochondria. It commits apoptosis by interacting with two mitochondrial proteins in host cells [37]. The Ser residue, but not the Asn residue, at position 66 of PB1-F2 is critical for the high pathogenicity of an H5N1 in mice [38]. The 1918 pandemic IVs carried the mutation of the Asn residue at position 66 to Ser in the PB1-F2 protein. The replacement of Ser with Asn attenuated the strong infectivity of the 1918 IVs, which pinpointed the PB1-F2 protein as a critical determinant of viral pathogenicity. PB1-F2 interacts with PB1 and affects the shuttling of this protein between the nuclei and cytoplasm [39], this shuttling ability might be affecting virulence. Thus, the shuttling ability seems to be critical in this context. IAVs attach to host cells via the binding of the hemagglutinin (HA) protein to the sialosaccharides of glycoproteins on the cell surface. The binding specificities of HAs in IVs derived from different host species are different. For example, HAs of human IAVs recognize sialic acid (SA)-α-2,6-Gal-terminated saccharides (α-2,6-SA) mainly, whereas HAs of avian IVs preferentially select to bind SA-α-2,3-Gal-terminated saccharides (α-2,3-SA). The horizontal avian-to-human transmission of IVs was abolished by mutations of two amino acids in HA that produced a switch in preferential binding from avian α-2,3-SA to human α-2,6-SA [40]. The pattern of virus attachment of the two subtypes of human IAVs (H3N2 and H1N1) and low pathogenic avian IVs (H5N9 and H6N1) was compared with the pattern of viral attachment of avian H5N1, which is highly pandemic [41]. Thus, the recognition pattern of IAVs to HAs might be critical for determining the degree of pathogenicity. However, the sugar-mediated binding specificity of IAVs to HAs varies according to specific viruses, which might hamper the development of new anti-IAV drugs.

In contrast to human IVs, the avian H5N1 virus binds mainly to the alveolar and bronchiolar epithelium, causing the damages in diffused alveolar epithelium as the primary hit. Viremia and extra-respiratory complications seem to be more common in infections with the avian H5N1 than with human IVs.

It has been reported that IV infection leads to the induction of oxidative stress or ROS damage and the development of clinical output. The direct clinical use of antioxidation drugs in IV-infected patients has not been reported. In humans, IAV infection increases the levels of metabolites such as 8-hydroxydeoxyguanosine, malondialdehyde, 2-isoprostane, 7-ketocholesterol, 7-beta-hydroxycholesterol, and carbonyl compounds in the plasma and urine. The levels of antioxidant enzymes, such as SOD and catalase; cytokines, such as IL-6, 1L-10, and TNF-α; and HSPs, such as HSP90 and HSP27 were also increased in H1N1-infected patients [24, 42]. Increased ROS, nitric oxide synthase 2 (iNOS2), and nitrotyrosine levels have been detected in patients and in a mouse model [43]. The results obtained from mouse models and cell lines infected with IVs demonstrated the production of enhanced levels of ROS, together with an imbalance of antioxidant protection [44, 45]. These models indicated the relevance of the redox homeostasis induced by IVs [46–50].

Antioxidation molecules ameliorate ROS damage in IV-infected host cells, whereas ROS enhance the pathogenic ability of IVs [26, 46]. To examine the role of oxygen free radicals in hosts, SOD conjugated with a copolymer of pyran was administered to mice, in an attempt to decay free radicals; this approach prevented infection with IV in these animals [51]. GSH inhibits the expression of viral matrix proteins, IV replication, and the production of virion particles. Furthermore, GSH suppresses the upregulation of Fas, caspase activation, and apoptosis in infected cells [52]. However, IV infection disrupts the redox balance by decreasing GSH production and promoting the propagation of the virus progeny, thus resulting in cell death [53]. The mechanism underlying the IV-induced downregulating of GSH remains unknown.

shRNA-mediated knockdown of Nrf2 decreased the expression of the Nrf2 protein in human nasal epithelial cells, whereas the upregulation of Nrf2 was strongly correlated with viral entry and its replication, indicating an inverse relationship between the levels of Nrf2 expression and susceptibility to IV infection [54].

**Fig. 1:**
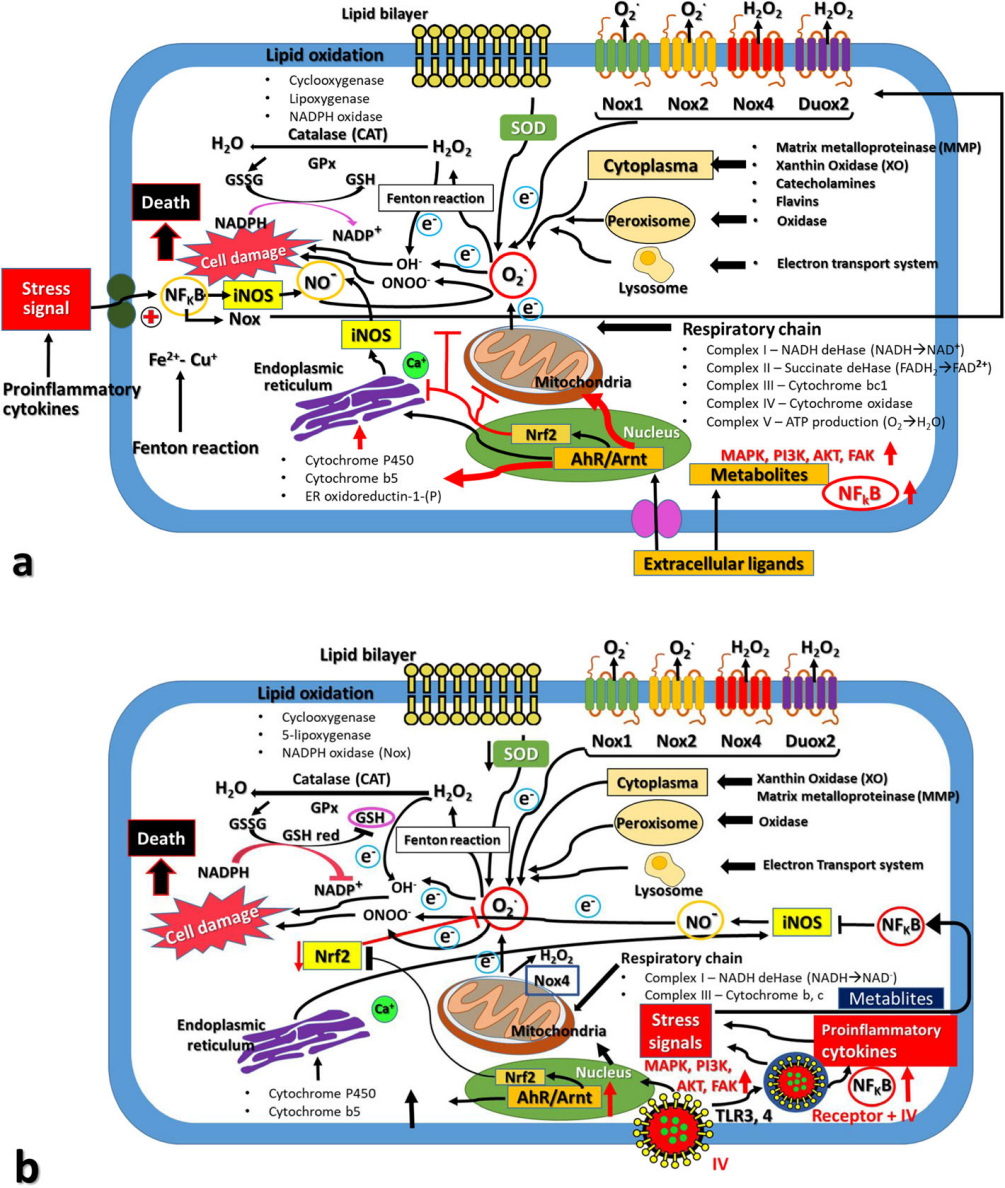
Schematic representation of ROS/RNS production and Redox control in response to stresses inducing reagents and influenza virus (IV) infection. Subcellular organelles and cellular components were affected by the oxidation and antioxidation responses in the case of stress inducers like phase I reagents and environmental hormones (Panel **a**) and infection of IVs (Panel **b**). The source of cellular ROS/RNS production mainly occurred in mitochondria, endoplasmic reticulum, lipid bilayer, organelle membranes, cellular lipids, DNA in the nuclei, and cellular oxidation enzymes and antioxidation enzymes such as superoxide dismutases (SODs), catalases (CATs), peroxidases (Prdxs), glutathione peroxidase (GPx) and glutaredoxins (GRs) and so on. In addition, the cellular signaling pathways such as NF-kB, MAPK, PI3K, AKT and iNOS signaling are also shown. After IVs infection, NOX produced superoxide (O_2_^-^) and dysfunction on the mitochondrial proteins. The defective mitochondrial proteins resulted in the leakage of electrons and superoxides from the mitochondria, as well as initiating cell death pathways by cytochrome c or permeability transition pore (PTP). The NF-kB produced many cytokines as well as inducible nitric oxide (NO) synthase (iNOS). This iNOS then produced nitric oxides (NO). The NO and O_2_^-^ reacted together to produce peroxynitrite (ONOO) which is a highly reactive compound to generate the protein nitration and damage of macromolecules. and viral mutations. Higher generation of O_2_^-^ resulted in the production of H_2_O_2_ by the catalytic activity of SOD. Uncontrolled production of H_2_O_2_ produced hydroxyl radicals (OH^-^) via reaction with metal cations, and these H2O2 and OH^-^ caused irreversible damages to cellular proteins, lipids, nucleic acids and so on. This Figure is a modified version of the ones published by Di Meo et al. [16] and Kohmich et al. [46].

The activation of the Nrf2/heme oxygenase 1 (HO-1) and Toll-like receptor (TLR)/mitogen activated protein kinase (MAPK)/nuclear factor kappa B (NF-κB) signaling pathways is involved in IV replication and IV-related pneumonia [55–57]. In some cases, the AhR also regulates redox genes, such as the *NQO1* gene, to maintain the ROS balance in host cells [58]. It is well known that the *NQO1* gene is regulated by the ARE, which is mediated by Nrf2 and the small Maf family of proteins [59]. In addition, the *NQO1* gene is also regulated by the AhR/Arnt complex, which binds to the Dioxin- responsive element in the *NQO1* promoter, in addition to the cytochrome p450 promoters, followed by the detoxification and blockage of the ROS production. Thus, we speculate that the binding of the AhR to the ligand after IV infection induces the oxidation of DNA, protein, and lipid components within cells to produce ROS at a higher level, thus leading to apoptosis and autophagy (**Fig.****1** [49, 60];).

In general, AhR induces the detoxifying enzyme cytochrome p450 and the phase II enzyme NQO1 or HO-1, to achieve redox regulation and maintain ROS balance; these molecules contain ARE *cis*-elements, which are controlled by Nrf2. Thus, the AhR–Nrf2 transcription factor battery plays a key role in the maintenance of ROS balance. In the case of IV infection, this theory might be adopted, as like the case of phase I ligand exposure [61, 62].

At the molecular level, IVs induce oxidative stress via AhR pathway in the cytoplasm, especially stresses in the endoplasmic reticulum (ER) and mitochondria, which are followed by the production of ROS. Simultaneously, the AhR transcription factor is translocated into the nucleus to activate the phase I target genes, which encode detoxification enzymes, several antioxidation enzymes, and immunoregulatory and allergy-related genes of antiviral immunity [10, 63, 64]. If the extent of ROS production surpasses the level of antioxidation, the ROS balance was abrogated, and the IV-infected cells commit to apoptosis and necrosis for cell death. If the ROS generation is cancelled by the antioxidation, the redox balance is remained to commit the resistance of host cells to the IV infection and the anti-viral immunity.

Cellular proliferation and apoptosis/necrosis, in addition to the immunity and allergy, are mostly dependent on the ROS balance [65]. Thus, ROS act as bifunctional reactors and exhibit both “good and bad” aspects in living cells, which might be similar in IV-infected host cells and the propagation of IV progeny [66]. These cellular events after IV infection were summarized in Fig. 1b.

### ROS, NOS, and RNS production in IV-infected cells

ROS are defined as chemically reactive species containing oxygen, and RNS are ROS that contain nitrogen instead of oxygen. Increased ROS production, the activation of iNOS2 for RNS production, and increased higher nitrotyrosine levels have been reported in IV-infected patients [12, 67]. Some of the sources of ROS in IV-infected host cells are summarized below. The generation of iNOS, NO, and 8-nitroguanosine lead to the IV-induced production of the superoxide anion [12, 16, 68].

#### PB1-F2 induces ROS production in host cells

PB1-F2 in influenza type A interacts with the adenine nucleotide translocator and the voltage-dependent anion channel 1, and inactivates matrix metalloproteinases, releases proapoptotic proteins, and induces cell death [69]. PB1-F2 is involved in the generation of mitochondrial ROS in alveolar epithelial cells by downregulating SOD1 [70]. In addition, H7N9 PB1-F2-induced ROS trigger inflammasome activation and IL-1β secretion, which is inhibited by Mitotempo, an inhibitor of mitochondrial ROS [71]. After viral infections, nucleotide-binding and oligomerization domain (NOD)-like receptor pyrin domain-containing-3 (NLRP3) inflammasomes are activated to induce pyroptosis, a cell death pathway that is inherently associated with inflammation via the activation of caspase-1 and the secretion of cytokines from infected cells [70, 71].

#### NOX and DUOX protein families

IV infection can induce cell death through viral PB1-F2, which targets mitochondria. Moreover, this cell death is conserved in IV type A, but not in IV type B [69]. In addition to the mitochondrial respiratory complex, ROS generated from NOX are involved in the pathogenesis of IV infection. The NOX family is found in cell and phagosome membranes, and comprises several members: NOX1 to NOX5, DUOX1, and DUOX2 [72]. The NOX family is expressed in cell-type-specific manner and is critical for the infection permissiveness of IV [72]. NOX1 is expressed in epithelial cells in the colon, placenta, prostate, and uterus, as well as in vascular smooth muscle cells, astrocytes, microglia, neural cells, and retinal pericytes. NOX2 is detected in cardiomyocytes, the endothelium and coronary microvascular endothelial cells, phagocytes, granulocytes in the umbilical vein, fibroblasts, hepatocytes, hematopoietic stem cells, neuronal cells, and vascular cells in skeletal muscles. NOX3 is expressed in the fetal kidney, liver, lung, and spleen and, at lower levels, in the adult colon and kidney. NOX4 is expressed in the eye, kidney, liver, lung, and ovary, as well as endothelial cells, fibroblasts, keratinocytes, mesangial cells, neurons, osteoclasts, and smooth muscle cells. NOX5 is expressed in mammary glands and the cerebrum, heart, kidney, liver, lung, lymphoid tissue, prostate, skeletal muscle, spleen, testis, and thymus, as well as vascular smooth muscle cells. Both DUOX1 and DUOX2 are detected in the colon, cecum, duodenum, floating colon, ileum, jejunum, pancreas, pancreatic islets, prostate, rectum, stomach, thyroid, sigmoidal colon, and the tracheal and bronchi epithelium [44, 72–74].

Upregulation of *NOX* and downregulation of *SOD1*, *SOD3*, *Nrf2*, and *CAT* were reported in H5N1-infected A549 cells [53]. Infection with H5N1 decreases *SOD1* promoter activity, whereas the overexpression of *SOD1* disrupts H5N1 virus replication in A549 cells.

#### NOX2 in lung alveolar epithelial cells

NOX2 seems to be involved in the production of ROS during IV infection [13, 75]. In a mouse model, Nox2-derived superoxide is critical for IV-induced pulmonary damage. *Nox2*^*–/–*^ knockout (KO) mice exhibited a milder airway inflammation and less apoptosis in the alveolar epithelium after IV infection than did wild-type mice [75]. The IV-mediated production of ROS and RNS was also decreased in *Nox2*^*–/–*^ KO mice [75]. In another study, the titer of active competent viruses and their associated inflammatory activities were decreased in these KO mice [72]. These findings suggest that NOX2 is a candidate for anti-ROS treatment to manage IV infection [75].

#### NOX4 in lung epithelial cells

NOX4 plays a role in ROS production in lung cancer and in IV-infected primary epithelial cells [44]. NOX4 induces both death and survival in tumor necrosis factor alpha (TNF-α)-challenged cerebral microvascular endothelial cells derived from newborn piglets. TNF-α-induced NOX4 activation causes oxidation-related cell death. Concomitantly, NOX4-derived ROS initiate the carbon monoxide (CO)-mediated survival of cells through the activation of heme oxygenase-2. CO prevents NOX4 activation, decreases oxidation, and ultimately promotes the survival of brain endothelial cells through the AKT signaling pathway. Moreover, CO inhibits TNF-α -induced ERK1/2 and p38 MAPK in an AKT-dependent manner. These findings suggest that NOX4 is a key element in the ROS-controlled survival of endothelial cells during TNF-α-mediated brain inflammation [76].

#### NOX1 in bronchoalveolar lavage fluid-derived fibroblasts

The production of superoxide anion in bronchoalveolar lavage fluid-derived fibroblasts from H3N2-infected *Nox1*^*–/–*^ KO mice was inhibited at day 7 after infection, although it did not differ from that observed in wild-type littermates at day 3 after infection (in the early stages of infection) [77]. Mice expressing an inactive Nox1 (Nox1*/γ) exhibited a higher survival rate after an influenza type A virus challenge than did control mice [78] [76]. The adaptive immune response was altered after the IV challenge in these mice, as shown by a decrease in the number of virus-specific CD8+ T cells in the lung, an increase in the number of virus-specific CD8+ T cells expressing CD127 (IL-7 receptor) in the lung, and draining of lymph nodes. These findings suggest that Nox1 negatively affects the early adaptive immune response to IV infection.

#### DUOX2 in the human nasal epithelium and mouse nasal mucosa

DUOX2 is another source of ROS production during IV infection [79, 80]. IV infection stimulates the induction of DUPX2/DUOXA2 and a moderate reduction of DUOX1 expression [77]. This DUOX2/DUOXA2 induction occurred after H1N1, but not after H3N2, infection [77]. In the human nasal epithelium and mouse nasal mucosa, DUOX2-induced ROS formation was triggered in airway cells via the type 1 and type 3 interferon (IFN) pathways, which induced the RIG-1-like receptor double-stranded RNA helicase enzyme (RIG-1) and the melanoma-associated differentiation gene 5 (MDA5) [81]. The increase in ROS production in mitochondria mediated by DUPOX2 or NOX2 is indicative of mitochondrial dysfunction [82].

### ROS in IV-induced tissue injury and cell death

Viral infection may alter redox control and pathophysiology and the antioxidation machineries. For example, Lin et al. reported that infection with H5N1 results in a higher level of ROS in A549 cells compared with infection with H1N1 and is accompanied by a significant reduction in the ratio of reduced glutathione (GSH) to oxidized glutathione (GSSG) [53].

#### IV-induced cell injury in the lung

In humans, IV infection causes a contagious respiratory disease in which many alterations of biological functions are induced, such as apoptosis and necrosis [83], autophagy [84], inflammation [85], lung injury (LI) [86], DNA damage and oxidation in host cells [87], lipid peroxidation [88], and ROS production in mitochondria [10].

Oxygen radicals are produced in the lungs of mice infected with IV [89] and ROS play a critical role in the acute lung injury (ALI) that occurs in mice infected with the highly pathogenic avian IV type A (H5N1) [90]. Moreover, in some cases, infection with H5N1 induced a high viral load and a strong proinflammatory reaction [36]. This action of H5N1 increases mortality and generates a more pronounced oxidative stress compared with other IVs, such as the human influenza A virus (H1N1).

Recent studies have reported that ROS production exerts a positive or negative effect during IV infection [91]. In the former case, viral infection can generate moderate ROS levels, which play a critical role in biological reactions with few cellular damage events. In contrast, excessive ROS are the major cause of LI. Downstream ROS targets, such as NOX1, NOX2, NOX4, and DUOX2, are involved in apoptotic cell death in the epithelium and ALI [46, 77]. After infection, IVs hijack the biological functions of host cells to enhance viral replication [24]. Accordingly, the imbalance between the redox control against IV and the production of excess ROS results in tissue damage [46].

#### Effect on the nervous system

IVs damage the central nervous system (CNS), leading to IV-associated encephalitis and encephalopathy [92]. Previous studies have suggested that IVs can infect astrocytes, which are the most abundant cells in the CNS and an integral part of the blood–brain barrier and induce a proinflammatory cytokine response and apoptosis [93, 94]. Lin et al. reported that human astrocytes induced the expression of CXCL9-11, NF-κB, and p38MAPK phosphorylation, as well as receptors of neurotransmitters, such as the melanocortin 2 receptor, cholinergic receptor nicotinic gamma subunit, purinergic receptor, gamma-aminobutyric acid (GABA) A receptor α1, and epidermal growth factor receptor 2, which are involved in synaptic transmission and CNS disorders [95]. More recently, it was reported that H5N1 bound and cleaved HA with a specificity for alpha-2,3-linked sialic acids, allowing the efficient binding of IVs and their efficient replication in CNS cells [96, 97]. Most of these reports are restricted to H5N1, except for one human H1N1 study [94, 95].

### IV-mediated ROS on cellular components

These IV-mediated functions, such as the effects on TLR family members, inflammation, matrix metalloproteinases (MMPs), and ER stress, in host cells are triggered, at least in part, by the regulation of Nrf2/ROS signaling and the signaling pathways of PI3K/AKT, p38/JNK MAPK, and NF-kB.

#### TLR family and membrane receptors

Human IV infections, such as H1N1 and H3N2, increase the expression of TLR family members, including TLR3, 7, 8, and 9; however, TLR2 and 4 are suppressed in this setting [98]. Another report showed that the expression of TLR2, 3, and 9 was correlated with H1N1 infection [99]. The upregulation of signaling molecules of IkB, P-MAPKs, and inflammatory cytokines (such as IL-6, sTNFR-1, MCP-1, CXCL10, and IFN gamma) is closely related with the upregulation of TLRs, MyD88, IRAK4, and TRAF6 and with human, avian, and swine IVs [57, 99–101].

*Tlr3*^*–/–*^ KO mice exhibit higher survival rates with lower viral titers, lower production of mediators of inflammation, and fewer pathological alterations in their lungs after IV infection than their wild-type counterparts [102, 103]. TLR7 is also required for the efficient replication of IVs [104]. The inhibitors of the TLR7/8–MyD88 axis possibly also inhibit IV replication and control proinflammatory cytokines and MMPs [105]. In contrast, the expression levels of TLR4 determine H1N1 entry and infection tropism via MyD88–p38 MAPK signaling [106]. Inactivated avian H5N1 can rapidly lead to the activation of the TLR4/TRIF/TRAF6/NF-κB axis [11]. *Tlr4*^*–/–*^ KO mice are refractory to H1N1-induced ALI, and a TLR4 antagonist decreases H1N1 viral titer and its lethality [107].

### ROS controls pattern-recognition receptors (PRRs)

The recognition of self and nonself in the innate immune reaction is carried out by pattern-recognition receptors (PRRs), which are the germline-encoded receptors located on the plasma membranes and within endosomes [108], that act as sensors to monitor infection. PRRs can sense an exogenous pathogen-associated molecular patterns (PAMPs) and endogenous danger-associated molecular patterns (DAMPs). DAMPs include ROS, heat-shock proteins, oxidized lipoproteins, and cholesterol. DAMPs can trigger innate inflammation by binding to PPRs, including TLRs, NOD-like receptors (NLRs), retinoic acid-inducible gene (RIG)-I-like receptors, and absent in melanoma (AIM) 2-like receptors (ALRs) [105]. The interaction with these receptors can amplify the inflammatory responses in the host cells. The innate immune receptors might modulate oxidative phosphorylation in mitochondrial function. Thus, oxidative stress responses can regulate PPRs for ROS/RNS mediated molecular targets.

In addition, the mucosal defenses of the lungs against IAVs can be followed by a single inhaled treatment comprising a synergistic combination of a TLR agonist (such as the diacylated lipopeptide ligand of TLR2/6, Pam2CSK4) and a CpG ligand for TLR9 (ODN362). These antiviral responses to viral burden attenuated the infectivity and enhanced survival potency via the protective responses afforded by ROS generation [109]. The interaction with these receptors can amplify the inflammatory responses in the host cells. The innate immune receptors might modulate oxidative phosphorylation in mitochondria. Thus, oxidative stress responses can regulate PPRs for ROS/RNS-mediated molecular targets.

#### Matrix metalloproteinases (MMPs)

H3N2 infection induces the expression of MMP-9, but not of MMP-2, in Vero cells. MMP-2 production is increased in Madin–Darby canine kidney (MDCK) cells. Thus, the induction of MMPs is dependent on the epithelial cell type [110]. The expression of MMP-9 is increased in the lungs of a mouse model of IAV infection [111]. The immunopathological response to IV strains via the production of MMP-9 was compared between the human IV viruses H1N1 and H3N2 in mice and revealed that H1N1 induces high mortality and severe lung changes with Gr1+ and CD11b+ cell infiltration and upregulation of CXCL6/GCP-2, CCL2/MCP-1, and the tissue inhibitor of metalloproteinase 1[112]. Infection with H1N1 upregulated the active and latent forms of MMP-9 in the lung and an inhibitor of MMP-2 or MMP-9 reduced in lung pathology partially. Both Gr-1+ and CD11b+ cells in H1N1-infected lungs produced ROS and RNS, indicating that MMP expression is controlled by oxidation and antioxidation. The human influenza type A virus induced the infiltration of neutrophils, which produced MMP-9 [113]. In contrast, MMP-9 production was not increased in human neutrophils after IV type A infection [114]. Thus, MMP-9 production in neutrophils is not controlled by IVs *per se*. Other cell types, such as macrophages, might regulate IV-mediated MMP-9 production. H1N1 induced the expression of the *MMP-9* gene and the cleavage of pro-MMP-2 into an active intermediate protein in human fetal membrane cells, resulting in the weakening of membrane integrity and the degradation of the extracellular matrix [115].

#### Potential interaction with inflammasomes

The inflammasome complex is involved in the inflammation-reaction-mediated sensing of microbial and viral components, and endogenous antigens [116]. At least two major types of inflammasomes, i.e., intracellular NLR family and the interferon-inducible protein absent in melanoma 2 (AIM2)-like receptor (ALR) family, have been identified [117]. Mitochondrial proteins are involved in the NOD–NLR family, and pyrin domain-containing 3 (NLRP3) inflammasome regulation [118]. Pathophysiological changes were observed in mitochondria, including the imbalance of mitochondrial dynamics, cytochrome c secretion, mitochondrial DNA damage, or ROS production, which can either activate or inhibit the NLRP3 inflammasome [119]. NLRP3 was activated by exogenous ligands such as PAMPs, including bacterial toxins, and by endogenous stimuli derived from the host, such as damage-associated molecular patterns. Upon IAV infection, the mitochondrial fusion protein mitofusin 2 can regulate the activation of the NLRP3 inflammasome [120]. The IAV PB1-F2 protein, which is translocated to mitochondria, can cause mitochondrial fission associated with the defective NLRP3 inflammasome [121]. However, there is not sufficient information regarding how dynamin-related protein 1 (DRP1)-mediated mitochondrial fission is generated in the innate immune signaling in IAV-infected cells. Swine IV (SIV) infection resulted in NLRP3 inflammasome activation, leading to IL-1β production in primary porcine alveolar macrophages (PAMs) [122]. Furthermore, upon SIV infection of PAMs, mitochondrial fission occurred through the RIPK1/DRP1 axis, which acted on the NLRP3 inflammasome-regulated production of IL-1β [123]. IAV produced endogenous ROS *via* NOX2-containing NOXs and mitochondria, to circumvent anti-IAV responses. These evolutionarily conserved processes were enhanced by glycolysis, the pentose phosphate pathway, and the tricarboxylic acid (TCA) cycle, which drove inflammation. These metabolites involved succinate, which stimulated the inflammation reaction through the ROS-mediated stabilization of hypoxia-inducible factor-1α, promoting production of interleukin-1β by the inflammasome. In addition, itaconate was investigated for its role as an anti-inflammatory and antioxidant metabolite of the TCA cycle [124]. NLRP3 inflammasome activation by decreased ROS through mitophagy might play a crucial role in berberine (BBR)-mediated alleviation of IV-induced inflammatory lesions [125].

#### Inflammation and ER stress

IV infection induces a robust production of cytokines, such as IFNs; interleukins (ILs); chemokines, such as CXCL10 and CCL5; tumor necrosis factors (TNFs); and ROS, which can promote the expression of inflammatory cytokines [126]. The generation of ROS is required in host cells after the activation of TLRs, which may be used by IVs to promote innate immunity functions in their hosts [11]. IVs trigger the production of proinflammatory cytokines/chemokines, such as CCL5/RANTES, CXCL10 (C-X-C motif chemokines), IL-1β, IL-6, IL-8, and TNF-α [127]. Some of these factors belong to the NF-κB signaling pathway, including IL-2, IL-6, IL-8, MIP1a, MCP-1, and RANTES [126]. These issues were reviewed by other authors [10, 128].

After IVs infect the host cells and the production of ROS/NOS surpasses the normal levels, events such as the production of oxidizing nitrogen oxides and peroxynitrite occur concomitantly. In turn, these events induce the oxidation or nitration of amino acid residues, lipid peroxidation, and DNA strand breaks, finally producing apoptotic signals in states of ER stress or of oxidative stress in mitochondria [129]. Thus, the generation of ROS is related to the cascades that commit the ER and mitochondria to apoptosis.

IV infection also induces ER stress and generates ROS in inflamed tissues [83, 129]. IVs induce proteasome-dependent ER-associated degradation through the inositol-requiring enzyme 1/x-box binding protein 1 (IRE1/XBP1) signaling pathway and commitment to downregulation of SOD1, thus allowing ROS accumulation. ROS-mediated JNK or the IRE1-mediated JNK1 contributes to the control of IV infection and propagation [129]. H1N1 PR8 infection of mouse tracheal epithelial cells increased the ER-stress-related ATF6 and the ER chaperone Erp57 [130]. IV infection induces caspase 12 to commit to apoptosis, as well as the production of TGF-β in IV-infected cells in a JNK1-dependent manner [130]. H1N1-infected mice exhibit induction of parenchymal lung inflammation, alveolar epithelial metaplasia, and ER stress. A lung epigenetic/transcriptome analysis demonstrated that mir-155 null mice recovered from IV infection and exhibited decreased lung inflammation and ER stress [131]. Protein disulfide isomerase 3 (PDIA3) interacts with IAV-HA, and this interaction is critical for the efficient oxidative folding of HA *in vitro*. PDIA3 is upregulated in IAV-infected mouse or human lung epithelial cells and interacts with IAV-HA directly. Treatment with a PDI inhibitor, LOC14, inhibited PDIA3 enzyme activity in lung epithelial cells, followed by the reduction of intradisulfide bond and subsequent oligomerization (maturation) of HA in both H1N1- and H3N2-infected lung epithelial cells [132]. IAV-mediated autophagy and unfolded protein response are connected to apoptosis in chronic stresses to regulate cellular homeostasis via CHOP and Beclin-1 [133].

#### Lipid oxidation

Malondialdehyde, F2-isoprostane, 7-ketocholesterol, and 7β-hydroxycholesterol have been identified as lipid alterations that are induced by IV-mediated ROS [24, 42]. IV infection induces oxidation, which is accompanied by an increase in the levels of lipid peroxidation in the presence of vitamin E, conjugated dienes, and total malondialdehyde [134]. IV-infected cells or animals exhibit increased oxidation of free radicals in unsaturated lipid chains in the host membranes, which decreases their permeability. In the absence of antioxidants, the cell membranes are severely damaged and ultimately exhibit severe pathological changes [135]. A/Aichi/2/68 IV infection increased the levels of lipid peroxidation (conjugated dienes and total malondialdehyde) and decreased the levels of endogenous natural antioxidant vitamin E. Supplementation of mice with exogenous vitamin E before viral infection protected the lungs and blood against lipid peroxidation damage [134].

#### DNA oxidation in host cells

DNA is the most common target of ROS, which they modify to give, for example, 8′-hydroxy-2′-deoxyguanosine (8-OHdG) at the 8-position of dG in the host DNA. 8-OHdG is produced via a transversion mutation of G to A in the host DNA and increases the risk of neoplasia [136].

### Cellular signaling mediated by IV-induced ROS

The triggering of these functions by IV is related to ROS generation and associated redox regulation.

#### Nrf2 signaling pathway

Redox homeostasis is maintained by cellular enzymes such as SODs, CAT, and GPx, which participate in antioxidant defense systems. IV infection can decrease the production of antioxidation targets, such as HO-1 and NQO1, SOD1 [53, 137], GR, CAT, and GPx1 [138] are downstream molecules of the Nrf2 pathway after IV infection. Thus, Nrf2 plays an important role in redox regulation upon IV infection [28, 49, 54, 139–141]. IVs activate the Nrf2/ARE antioxidation pathway via Nrf2, followed by the transcriptional activation of Nrf2 target genes, such as *HO-1* and *HMOX1* [140, 142].

The highly pathogenic avian H5N1 IV reduces the levels of phosphorylated Nrf2 in the nucleus and downregulates the expression of fibronectin to a greater extent than does the human H1N1 IV [143]. Several studies found no changes in the levels of SOD in IV-infected cells [135]; however, other studies found a contradictory lower level of SOD1 caused by protease degradation [52, 144]. Increased expression of SOD1 was reported in patients with asymptomatic IV infection [145]. Decreased levels of SOD1 have been found in children infected with H1N1 [146]. Therefore, whether SOD1 is a marker of IV infection remains uncertain.

Similar controversial findings have been reported for other antioxidant enzymes, such as CAT and indolamine-2,3-dioxygenase (IDO). IDO scavenges superoxide anion for oxidation or for converting tryptophan into kynurenine [147]. The IDO level is unaltered and the CAT levels are reduced in IV-infected cells in vitro [135]. In contrast, in infected mice, both IDO and HO-1 are induced, and CAT is unchanged [148]. Cat- peroxiredoxin-6 (Prdx6-)-deficient mice infected with H1N1 exhibit depletion of IV-permissive bronchial Clara cells and/or alveolar type 2 cells [142]. Similar reports showed the induction of other enzymes, such as GPX3 and HO-1. Other Nrf family members, such as Nrf1, bind to the ARE in the promoter regions of redox-related genes, although to a lesser extent than that observed for Nrf2. In contrast, Nrf3 does not behave similarly [149].

#### The p38 MAPK signaling pathway

p38 MAPK plays a vital role in cell proliferation, differentiation, development, and death. For example, phosphorylated p38 is translocated into the cell nucleus and upregulates cytokines/chemokines under oxidative stress. In Bcl-2+ cells (MDCK introduced by Bcl-2), p38MAPK interacts with Bcl-2 and colocalizes in the cytoplasm, and both Bcl-2 phosphorylation and apoptosis are decreased by specific p38MAPK activity. In contrast, in Bcl-2-negative (*Bcl-2*^*−/−*^) MDCK cells, which are fully permissive to viral infection, p38MAPK activity is present in nucleus predominantly, and its inhibition reduces the traffic of vRNP and the phosphorylation of viral nucleoproteins, suggesting that p38MAPK contributes to the regulation of vRNP export and viral replication [150]. Amatore et al. reported that IV infection upregulates NOX4 and that the NOX4-derived ROS activate p38 and ERK1-2 MAPK, which in turn promote the nuclear export of vRNP and consequently lead to viral replication [44]. Thus, p38MAPK might be related to the vRNP export by phosphorylation and viral replication. Curcumin inactivates IAV directly, and inhibits IAV adsorption and replication; moreover, its inhibition of IAV replication may occur through the Nrf2 signal, to inhibit the IAV-induced activation of the TLR2/4, p38/JNK MAPK, and NF-κB pathways. Thus, inactivation of p38/JNK MAPK, NF-kB and TLR2/4 exerted anti-influenza virus effects. p38/ JNK MAPK is a critical mediator of oxidation-induced apoptosis, to increase ROS and COX-2 production [151].

#### The NF-κB signaling pathway

NF-κB plays a key role in the activation of the immune system. The NF-κB complex comprises five proteins, namely Rel A (p65), c-Rel, Rel B, p50, and p52. The NF-κB p50/p65 heterodimer associated with IκBα is related to the outcome of oxidative stress [152, 153]. After phosphorylation of p65 at Ser 276, NF-κB antagonizes Nrf2 and suppresses the transcription of ARE-dependent genes by recruiting histone deacetylase 3 to the ARE [153]. Thus, inhibition of NF-κB activity may benefit Nrf2-mediated antioxidation and the suppression of IV-induced inflammation.

Cells treated with the focal adhesion kinase (FAK) inhibitor Y15 or expressing dominant-negative FAK kinase show a reduction in the H1N1-induced NF-kB promoter activity. The nuclear localization of NF-kB is reduced in cells expressing dominant-negative FAK kinase [154]. The human parainfluenza virus type 2 phosphoprotein (P) is a key component of viral polymerase. The P gene encodes both P and accessory V proteins via specific gene editing. Moreover, the nucleocytoplasmic shuttling of the P protein appears to be important for efficient viral polymerase activity [155].

The H3K79 methylation is critical for IV replication. The downregulation of the H3K79 methylase Dot1L causes a decrease in the nuclear localization of the NF-kB complex, to reduce the antiviral response [156].

#### The PI3K/AKT signaling pathways

IVs can modulate several oxidative-stress- and redox-activated signaling pathways, such as those involving NF-κB, MAPK, and PI3K/AKT [150, 157–159], to promote viral replication and pathogenesis [32, 150, 160–162]. Therefore, the modulation of these signaling pathways may attenuate IV-induced pulmonary damage.

### Activation of AhR augments IV virulence

TCDD-treated and IV-infected mice exhibit activation of AhR in the lungs and a decrease in survival, which suggests a relationship between the susceptibility to viral respiratory infections and exposure to environmental toxin ligands [32]. In this model, increased iNOS levels in endothelial cells of virus-infected mice and an increased number of neutrophils around pneumocytes are observed after AhR activation, which requires a nuclear transport signal and intact DNA-binding domains within AhR [33]. The activation of the AhR, which occurs via kynurenine mediation, regulates the production of IFNβ negatively after IV infection, which allows virus propagation [163]. IV infection can increase kynurenine production by upregulating the expression of indoleamine-2,3-dioxygenase (IDO1), which is a key enzyme in the kynurenine biosynthesis pathway [164].

In addition to the increased pulmonary neutrophilia and iNOS levels resulting from IV infection, mice treated with TCDD, which activates AhR only transiently, exhibit a diminished IV-specific CD8^+^ T-cell response [60]. This suggests that the prolonged AhR activation induced by the environmental pollutant TCDD correlates with increased respiratory IAV infection. Furthermore, Boule et al. [165] compared the effects of four AhR agonists, TCDD, 3,3′,4,4′,5-pentachlorobiphenyl-126 (PCB126) 2-(1*H*-indol-3-ylcarbonyl)-4-thiazolecarboxylic acid methyl ester (ITE), and FICZ, on the immune response in mice infected by IVs. Treatment with TCDD, PCB, and ITE decreased the virus-specific IgM/IgG levels and the number of helper T cells and CD8^+^ cytotoxic T cells but increased the number of regulatory T cells. However, FICZ alone decreased the levels of virus-specific IgG and the CD8^+^ T-cell response and increased the number of helper T cells. These studies suggest that harnessing AhR activity is critical for modulating the host cell immunity to IV infection. Dendritic cells (DCs) were another target [166]. Gene expression studies revealed changes in Lrp1, Itgam, and Fcgr1 expression, as well as alterations in genes that regulate the migration of DCs, and antigen processing/presentation disrupted by inappropriate AhR signaling during development. These studies suggest the importance of the AhR activation during development in DCs. Franhini et al. reported the genome-wide transcription map of AhR targets genes induced by influenza virus H3N2 in dendritic cells and found that the lectin receptor *Cd209a* (DC-SIGN) and chemokine the *Ccl17* are novel AhR target genes [167].

Regarding ROS production and AhR activity *in vivo*, although singlet molecular oxygen (^1^O_2_) is not fully characterized in mammals, its role is well established in plants, bacteria, and fungi. Although the mammalian enzyme myeloperoxidase mediates the production of ^1^O_2_, its physiological role in mammals (other than photosensitization of the skin by the UVA component of solar radiation) has not been established [168]. A recent report by Stanley et al. [169] showed that ^1^O_2_ plays a critical role in redox regulation in atrial relaxation and in controlling blood pressure in mammals during inflammation accompanied by endothelial IDO1 expression. Thus, ^1^O_2_ is an important ROS in the fields of biology and medicine.

### Possible anti-influenza therapies

During the IV infection, the cellular metabolism of host cells could be affected, leading to a dysregulation of redox homeostasis. IV viruses induce oxidative stresses via the increase in ROS generation and the alteration of ROS scavenging systems. As part of the antioxidation defense, selenoproteins, such as GPXs and thioredoxin reductases (TXNRDs), which are present in the ER, play a critical role in controlling oxidative stress [170]. Antioxidant therapies have been proposed to decrease viral load and to counteract the lung injuries caused by the overproduction of ROS induced by the viruses [171]. Some antioxidants are effective in this protection against infection through the Nrf2 pathway [49]. Antioxidant genes, which can be upregulated by Nrf2, play a critical role in the elimination of ROS/RNS; therefore, enhancement of Nrf2 activity and inhibition of AhR activity have been proposed as approaches to ameliorate the IV-associated pathology. For example, the downstream target of Nrf2, SOD conjugated with a copolymer of pyran, was administered in an attempt to decay free radicals; this approach prevented infection with IV in mice [51]. In this section, antioxidation drugs aimed at inhibiting ROS levels will be reviewed as potential therapeutics for IV infection.

#### Inhibition of AhR activity

AhR activation during IV infection disrupts host immunity and causes increased lung inflammation and mortality in mice [42, 64, 165, 172]. The suppression of AhR activity is assumed to attenuate IV-induced lung damage. The level of IV-induced IFNβ is increased in AhR-deficient cells and mice, thus leading to the suppression of viral replication [64]. Several AhR antagonists, such as CH-223191 and Stem Regenin 1, have been identified; however, their therapeutic value against IV-infection-induced LI is unclear. Because AhR responds differentially to diverse intrinsic and extrinsic ligands and affects multiple types of immune cells [172], a careful examination of the advantages and disadvantages of these AhR antagonists is required to assess their value in the treatment of IV infection.

#### N-acetyl l-cysteine (NAC)

NAC is a precursor of intracellular cysteine and GSH in mammals. NAC contributes to the resistance against IV infection through mechanisms that include the inhibition of IV replication, the production of proinflammatory cytokines, and the prevention of IV-induced apoptosis [173–176]. NAC suppresses viral replication and the expression of IV-mediated inflammatory factors, such as TNF-α, IL-6, and IL-1β, and then suppresses the inflammation induced by the highly pathogenic avian H5V1 virus [174]. In addition, cellular damage in the lungs suppresses TLR4 [174]. However, Garigliany et al. insisted that the effect of NAC is strain dependent and that this molecule lacks inhibitory activity against some IVs. NAC is effective against A/PR/8 and H5N1, but not against murinized swine H1N1 IV [177]. The synergistic use of NAC and antiviral drugs as a combination therapy provides effective protection against IV infection in mice [173, 175].

#### Glutathione

The antiviral activity of GSH may be involved in inhibiting the synthesis of viral proteins [178]. A higher level of GSH might interfere with the formation of disulfide bonds, thus preventing the correct folding of viral hemagglutinin (HA), followed by the alteration of its transport and its insertion into host cell membranes [179]. Furthermore, a derivative of GSH, *N*-butanoyl glutathione (GSH-C4), which is a cell-permeable chemical compound [180], diminishes IV replication by maintaining the immature monomeric HA in the ER and inhibiting the targeting of mature membrane glycoproteins, which is achieved via an increase in GSH levels [180]. GSH-C4 affects the inflammatory response via NF-kB signaling and improves the Th1 response against IV in old mice [181, 182].

**Fig. 2:**
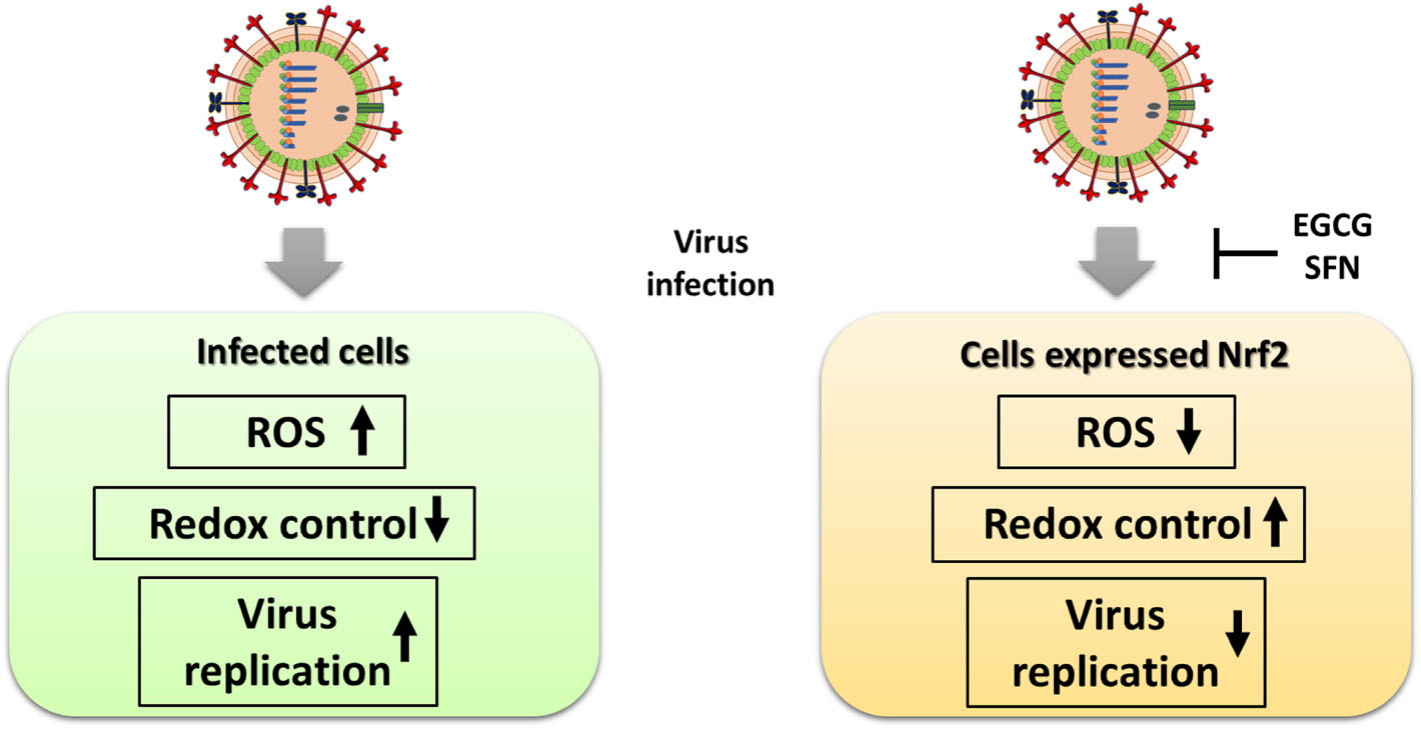
Model of infection by influenza viruses of normal host cells and cells overexpressing Nrf2. Infection by IVs led to the production of reactive oxygen species (ROS). Redox control against ROS and viral replication are illustrated. The addition of sulforaphane (SFN) or epigallocatechin gallate (EGCG) inhibits the viral infection of IV via activation of the antioxidation pathway [54].

GSH inhibits the viral apoptosis induced by IVs and the production of IV particles. It also depresses the expression of viral matrix proteins, caspase cascades, and Fas induction [52]. Moreover, nutritional supplements that induce GSH may afford resistance to the major pathogenic processes of H5N1 [183]. Bakuchiol, a phenolic isoprenoid, activates the Nrf2 pathway and blocks IV infection, which suggests that bakuchiol has antiviral activity [28]. Overexpression of Nrf2 or the addition of sulforaphane and EGCG decreases the replication of, and protein synthesis in, IVs [54] (**Fig.****2**). Moreover, Nrf2 knockdown increases the entry and replication of IVs and enhances IV-induced pulmonary cell injury [54, 141]. Nrf2 is also a factor in the outcome of IV-infected mice after exposure to cigarette smoke [141]. Nrf2-deficient mice exhibit more severe bronchial inflammation, permeability damage in the lungs, mucus hypersecretion, and higher mortality rates after IV infection and cigarette smoke exposure than do wild-type mice [141]. Taken together, these results suggest that the Nrf2-mediated antioxidant pathway plays a critical role in suppressing IV-induced LI under oxidative conditions, such as cigarette smoke exposure [48, 141, 182–184].

### Other small molecules

Many compounds that are traditionally regarded as antioxidants are really electrophiles, and their actions involve the activation of redox pathways through Nrf2 signaling, rather than the scavenging of ROS [185]. Isoprenoid phenols (e.g., baicalein and biochanin) prevent the replication of the highly pathogenic avian H5N1 virus by repressing ROS production [186]. EGCG and catechin exhibit an antiviral activity that involves antioxidation [187, 188]. Quercetin decreases the production of superoxide in alveolar macrophages during IV infection [88] and exerts an antiviral activity by inhibiting HA2 during IV infection [189]. A bioflavonoid isolated from *Garcinia kola* seeds (kolaviron) exhibits strong anti-IV activity via an antioxidative pathway [190].

#### Nontargeted inhibition (NF-κB, p38 MAPK, and PI3K/AKT inhibitors)

The effects of the dietary flavonoid kaempferol on H9N2-mediated acute LI included the repression of oxidative stress and inflammatory responses via the downregulation of NF-κB [57]. Kaempferol inhibits the NF-κB and MAPK pathways, leading to an increase in SOD activity, the attenuation of ROS levels, and H9N2-induced acute LI [57]. Some ROS scavengers, such as polyphenols, may modulate the NF-κB and MAPK pathways and upregulate GSH biosynthesis through Nrf2 activation [154, 172, 190–194]. The immune-regulatory properties of NAC and IV-induced pneumonia are related to the inhibition of the p38 MAPK pathway [195]. Thus, NF-κB activation and the p38 MAPK, pI3K/AKT, and PKC/PKR pathways may serve as biomarkers of oxidative stress [192, 196]. M2 ion channel inhibitors, such as oseltamivir/amantadine (AM)-modified selenium nanoparticles (SeNPs; Se@AM), inhibit the H1N1-induced apoptosis of host cells through ROS-mediated AKT signaling pathways [161].

## Conclusions

**Fig. 3:**
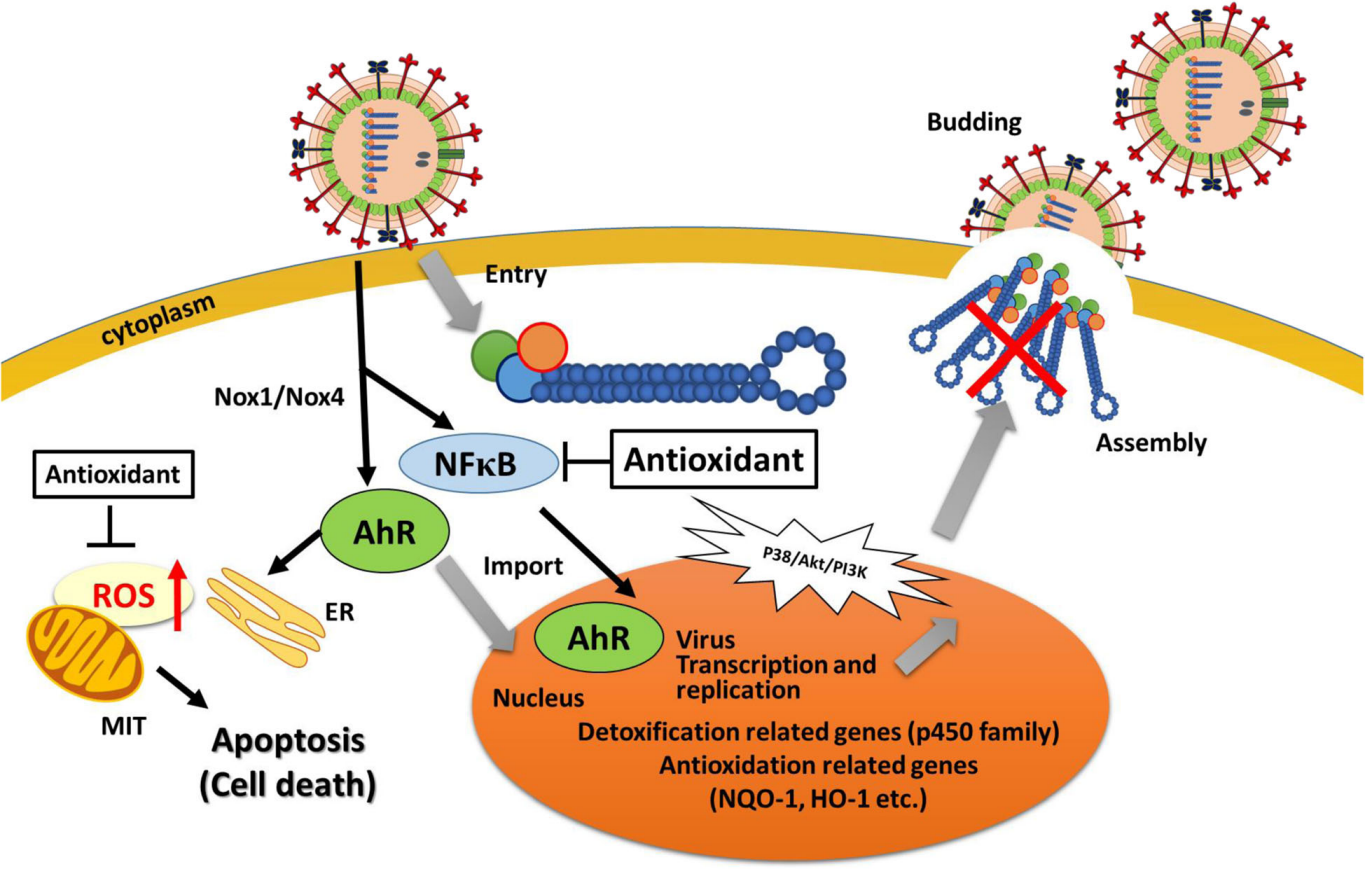
Schematic model of the mechanism of ROS production and antioxidation during infection with IVs. During the life cycle of the IV, infection causes oxidative stress reactions in host cells, which leads to induction of Nox1/Nox4 and AhR and the production of ROS, followed by replication of the viruses. Nrf2 and other signaling molecules, such as p38, AKT, and PI3K, are also involved in ROS stress and redox control. Oxidative stress also induces the translocation of the AhR transcription factor to the ER and mitochondria (MIT), which increases ROS production. The antioxidation against ROS by the Nrf2 transcription factor helps to prevent cell damage during the initial phase; however, the excess of ROS causes apoptosis and other types of cellular death in infected host cells. The life cycle of IVs is summarized and the possible targets of drugs to treat IV infection are also indicated.

The pathophysiology of IV infection is related, at least in part, with the imbalance between oxidation and antioxidation systems, as well as with the state of AhR activation (**Fig.****3**). In this review, we presented several examples of the effect of the IV–host interactions on the intracellular redox and ROS states. Based on this knowledge, several potential therapeutics for the treatment or prevention of IV infection are presented (**Tables****1****and****2**). Heaton et al. [256] reported a CRISPR activation screening that aimed to identify a pan-avian IV-inhibitor host factor; those authors isolated the glycosyltransferase B4GALNT2, which can modify glycans containing α-2,3-linked sialic acids. However, most of these proposed therapeutic strategies require validation using animal models of IV infection and human clinical trials. Although many studies have uncovered the multifaceted roles of the cellular redox system and of ROS activity in IV-infection-induced lung inflammation and injury, several questions remain unanswered that are important for illustrating the precise IV–host interaction and that await further investigation. For example, both oxidative stress and virus replication result in chromatin remodeling, which largely affects gene expression, including that of Nrf2- and AhR-regulated genes, and determines the outcome of the virus–host interaction. Understanding the precise crosstalk between multiple chromatin modifiers, such as histone acetyltransferases/deacetylases and methyltransferases/demethylases, and the transcription factors Nrf2 and AhR upon IV infection is required to predict the consequences of viral infection accurately. Accordingly, a more specific and effective intervention for IV infection may be developed by targeting either ROS homeostasis or chromatin modifiers [257, 258]. In conclusion, this review presents the notion that, in addition to viral components, the therapeutic treatment of IV infection may be achieved by targeting virus-induced ROS and redox-associated cellular responses, which may suppress IV propagation and reduce adverse inflammation in the host.

**Table 1:** Drugs and small molecules against ROS that prevent infection with influenza viruses. **[Against ROS]**

Thiol compounds and prodrugs	Effect on influenza virus infection	References
N-acetyl-L-cysteine (NAC)	• Reduction of the cell population at the G0/G1 phase • Reduction of pro-inflammatory molecule	Geiler et al. [174] Ghezzi et al. [175] Wu et al. [197] Zhang et al .[176] Garigliany et al. [177]
Glutathione (GSH)	• Affects viral mRNA export and decreases the expression of late viral proteins • Inhibition of caspase activation and Fas upregulation	Nencioni et al .[198] Cai et al. [52]
GSH-C4	• Inhibition of influenza virus HA maturation • Inhibition of influenza virus replication and Th1 immune cells induction	Sgarbanti et al. [180] Amatore et al. [44]
PDTC (pyrrolidine dithiocarbamate)	• Decrease in viral RNA synthesis • Inhibition of apoptosis	Uchide et al. [199] Qi et al. [200]
Hydroxyl antioxidants	Effect on influenza virus infection	References
NDGA (Nordihydroguaiaretic acid)	• Inhibition of apoptotic DNA fragmentation and virus proliferation	Uchide et al. [201]
Thujaplicin	• Inhibition of apoptosis, virus replication and release from the infected cells	Miyamoto et al. [202] Uchide et al .[203]
Resveratrol/ Vitisin A (tetramer of resveratrol)	• Inhibition of the nuclear–cytoplasmic translocation of vRNP • Downregulation of viral proteins • Inhibition of protein kinase C activity • Inhibition of virus-induced RANTES production, to decrease of the virus-stimulated phosphorylation of Akt and STAT1	Palamara et al. [204] Huang et al. [205] Uchide et al .[203]
Ambroxol	• Stimulation of the release of pulmonary surfactants, mucus protease inhibitor, IgA, and IgG • Suppression of the release of cytokines, TNF-α IFN-γ, and interleukin-12	Yang et al. [206] Uchide et al. [203]
Ascorbic acid	• Inhibition of the entry of viruses • Increase in the production of IFN-α/β at the initial stage of infection • Inhibition of excessive CORT synthesis	Wang et al. [207] Kim et al. [208] Cai et al. [209] Kim et al. [210]
Tert-buthylhydroquinone (tBHQ)	• Inhibiting of ROS production and increase antioxidation	Antanasijevic et al. [211, 212]
Monoacetylcodine (MAC) + Curcumin	• Inhibition NA activity • Inhibition IAV infection better then curcumin only	Richart et al. [213]
Curcumin + Resveratrol	• Scavenging of H2O2, HON, and ROON • Inhibition of TLR 2/4, p38MAPK, and NFkB	Sharma et al. [214] Barzeger et al. [215] Dai et al. [216]
Emodin (1,3,8-trihydroxy-6-methyl anthraquinone)	• Inhibition of IA replication, IV pneumonia • Inhibition of TLR 4, p38/JNK, and NFkB	Dai et al. [217]
Oxymatrine (OMT); C15H24N2O20, imunosuppressive reagent	• Antioxidant • Suppression of inflammation and viral infections • Hepatoprotective and immunosuppressive • Inducer of TLR4, p38 MAPK, NFkB, and PI3K/AkT	Dai et al. [56]
Aurantiamide acetate (E17)	• Strong anti-inflammatory and antiviral effects	Zhou et al. [218]
4-PBA (4-phenyl butyrate)	• Inhibitor of ER stress	Jung et al. [129]
Kaempferol	• Inhibition of TLR4/MyD88-mediated signaling of NFkB and MAPK	Zhang et al. [57]
Apocynin	• Inhibitor of NOX2 • Inhibition of ROS and IV-induced cytokine production	Ye et al. [45]
Flavonoids	Effect on influenza virus infection	References
Dianthus (quercetin 3; isorhamnetin 3)	• Binding to IV polymerase membrane glycoproteins ROS inhibitor	Kim et al. [219]
Quercetin Quercetin 3-glucoside	• Protecting low-density lipoprotein against oxidation • Antithrombic, antivirus, and anti-inflammatory effcets • Supression of ROS	Formica et al. [220] Nile et al. [221]
Polyphenol	Effect on influenza virus infection	References
Chlorogenic acid	• Antivirus and antihypertension effects • Protection of dopaminergic neurons against neuroinflammation	Zhao et al. [222] Shen et al. [223] Ding et al. [224]
Bakuchiol	• Activation of Nrf2 pathway	Shoji et al. [28]
Chemicals	Effect on influenza virus infection	References
Poly (aniline-co-pyrrole) polymerized nanoregulators (PASomes) with mPEG-b-pPhe (methoxy polyethylene glycol-block-polyphenylalanine copolymer)	• Inhibition of ROS production • Inhibition of viral replication and cell death	Kim et al. [225]
Cholesterol conjugated gp91 of NOX2 oxidase gp91phox sequence linked to the human immunodeficiency virus-tat peptide (Cgp91de-TAT)	• Inhibitor of NOX2 oxidase • Inhibitor of ROS and inflammation	To et al. [226]
Ectonucleotide pyrophosphatase/phosphodiesterase 1 (ENPP-1)	• Inhibition of HO-1 • Inhibition of H9N2 proliferation	Qi et al. [227]
Genes	Effect on influenza virus infection	References
Bax Inhibitor-1	• Inhibiting ROS mdiated cell death and augmented HO-1 • H9N2-NS1 induced ROS and apoptosis	Hosain et al. [228] Qi et al. [200]

**Table 2:** Drugs and small molecules against influenza virus that prevent infection with influenza viruses. **[Against Influenza viruses]**

Hydroxyl antioxidants	Effect on influenza virus infection	References
Atorvastatin (Lipitor)	Inhibition of HMG-CoA reductase	Episcopio et al. [229]
Clarithromycin (Biaxin)	Inhibition of MCP-1 and MMP-9, Increases of IL6 and IFNgamma	Takahashi et al. [230]
Flavonoids	Effect on influenza virus infection	References
5,7,4’-Trihydroxy-8-methoxyflavone	Inhibition of virus fusion with endosome/lysosome membranes	Nagai et al. [231–235]
Catechins	Inhibition of HA and NA activity Inhibition of viral endonuclease activity	Song et al. [188] Kazuhara et al. [236]
Quercetin 3-rhamnoside	Reduction of the cytopathic effect (CPE)	Choi et al. [237]
Isoquercetin	Decrease in viral titers	Kim et al. [219]
Oligonol (+NAC)	Inhibition of nuclear export of vRNP	Gangehei et al .[238]
Viral enzymes and membrane proteins as targets	Effect on influenza virus infection	References
Amantadine	Inhibitor of the matrix protein M2	Pica and Palese [239]
	Selenium nanoparticles with amantadine ROS-mediated AKT phosphorylation signal against H1N1	Nabar et al. [240]
	Selenium nanoparticles with ribavirin RNA polymerase inhibitor	Lin et al. [241] Li et al. [161]
	Activation of the caspase-3 apoptotic pathway against H1N1	Li et al. [161] Lin el al [241].
Prion	Protects mice from lethal infection with IAV Reduce the ROS in infected lung	Chida et al. [242]
NS1	H9N2-NS1 induced ROS and apoptosis	Qi et al. [200]
Oseltamivir and zanamivir	Inhibitor of neuraminidase (NA)	Kode et al. [243]
Laninamivir	Inhibitor of HA	Tomozawa et al. [244]
Peramivir	Inhibitor of HA	Scott et al. [245]
Rimantadine	Inhibitor of M2 ion channel	Alves Galvão et al. [246]
NMS-873	Inhibitor of p97 (AAA+ ATPase, VCP) Oseltamivir resistant strain specific	Zhang et al. [247]
Baloxavir marboxil	Cap-dependent endonuclease inhibitor	O’Hanlon et al. [248]
1,3-dihydorxy-6-benzo[C] chromone D715-2441 + zanamivir	PB2 Inhibitor Binding to PBcap	Liu et al. [249]
Salinomycin + oseltamivir (OSV-P)	M2 channel blocker Inhibition of HA	Jang et al., [250]
10e (Am2-S31N blocker)	Proton channel blocker and M2 blocker	Hu et al. [251]
Favipiravir	PB1 inhibitor	Goldhill et al. [252]
Triple combination of Zanamivir + Clarithromycin + Flufenamic acid (FFA)	Inhibitor of HA	Lee et al. [253]
Others	Effect on influenza virus infection	References
Single-walled carbon nanotubes (SWCNTs)	Increasing of H1N1 viral titer	Chen et al. [254] Sanpui et al. [255]
Umifenovir	Interact with HA to inhibit HA	Chida et al. [242]

## Data Availability

Not applicable.

## Abbreviations

AhR: Aryl hydrocarbon receptor
AIM: Absence in melanoma
ALR: AIM2-like receptor
ALI: Acute lung injury
ARE: Antioxidant response element
BBR: Berberine
CAT: Catalase
CO: Carbon monoxide
CSN: Central nervous system
CYP: Cytochrome p450
DAMP: Danger-associated molecular pattern
DC: Dendric cells
DRP1: Dynamin-related protein 1
Duox: Dual oxidase
EGCG: Epigallocatechin gallate
ER: Endoplasmic reticulum
GABA: Gamma-aminobutyric acid
GPx: Glutathione peroxidase
FAK: Focal adhesion kinase
Fe-S: Iron-sulfur
GR: Glutaredoxin
GSH: Glutathione
GSH-C4: N-butanoyl glutathione
GSSG: Glutathione disulfide
HA: Hemagglutinin
HO-1: Heme oxygenase-1
H_2_O_2_: Hydrogen peroxide
8-OHdG: 8'-hydroxy-2'-deoxyguanosine
OH: Hydroxyl radicals
H_2_S: Hydrogen sulfide
HS: Sulfanyl radical
IAV: Influenza A virus
IDO: Indoleamine-2,3-dooxygenase
IFN: Interferon
iNOS: Inducible nitric oxide synthetase
IRE1: Inositol-requiring enzyme 1
IV: Influenza virus
Keap1: Kelch-like ECH-associated protein 1
LI: Lung injury
MAPK: Mitogen activated protein kinase
MDA5: Melanoma-associated differentiation gene 5
MDCK: Madin-Darby canine kidney cells
MIT: Mitochondria
MMP: Matrix metalloproteinase
NAC: N-acetyl L-cysteine
NADPH: Nicotine adenine dinucleotide phosphate
NFkB: Nuclear factor kappa B
NLRP3: NOD-like receptor pyrin domain-containing-3 (NLRP3)
NO: Nitric oxide
NOD: Nucleotide-binding and oligomerization domain
NOX: Nicotine adenine dinucleotide phosphate oxidase
NP: Nucleoprotein
Nrf2: Nuclear factor E2-related factor 2
NQO1: NADPH quinone oxidoreductase 1
O_2_^–^: Superoxide anion
PAMP: Pathogen-associated molecular pattern
PAN: Porcine alveolar macrophage
PB1: Polymerase basic protein 1
PB2: Polymerase basic protein 2
PDIA 3: Protein disulfide isomerase 3
Prdx: Peroxiredoxin
Prr: Pattern-recognition receptor
RIG: Retinoic acid-inducible gene
SOD: Superoxide dismutase
RNS: Reactive nitrogen species
ROS: Reactive oxygen species
SA: Sialic acid
SFN: Sulforaphane
SIV: Swine IV
TCA: Tricarboxylic acid
TCDD: 2,3,7,8-Tetrachlorodibenzodioxin
TLR: Toll-like receptor
TNF*a*: Tumor necrosis factor α
TXNRD: Thioredoxin reductase
vRNP: viral ribonucleoprotein
XBP1: X-box binding protein 1
XO: Xanthine oxidase
